# Supplementary material for: Urea Decomposition Mechanism by Dinuclear Nickel Complexes
Source: Molecules. 2023 Feb 9;28(4):1659. doi: 10.3390/molecules28041659 (PMC9964345; doi:10.3390/molecules28041659)
Supplement: Supplementary file 1 [file molecules-28-01659-s001.zip › molecules-2156005-supplementary.pdf]

Support Information

Urea decomposition mechanism by dinuclear nickel complexes

Christian O. Martin [a, b], Leticia K. Sebastiany [a], Alejandro Lopez-Castillo [a], Rafael S. Freitas [c], Leandro H. Andrade[d], Henrique E. Toma *[d], Caterina G. C. Marques Netto* [a]

[a] Department of Chemistry, Universidade Federal de São Carlos, Rod. Washington Luiz, s/n, km 235- São Carlos/SP – Brazil
[b] Department of Chemical Engineering, Universidade Federal de São Carlos, Rod. Washington Luiz, s/n, km 235- São Carlos/SP – Brazil
[c] Institute of Physics, Universidade de São Paulo, Rua do Matão, 1371, Cidade Universitária-SP/Brazil
[d] Institute of Chemistry, Universidade de São Paulo, Av. Prof. Lineu Prestes 748, Cidade Universitária-SP/Brazil
*Correspondence: HET: henetoma@iq.usp.br; CGCMN: caterina@ufscar,br

Characterization of [Ni_2L(OAc)] and [Ni_2L(Cl)(Et_3N)$_2$] complexes

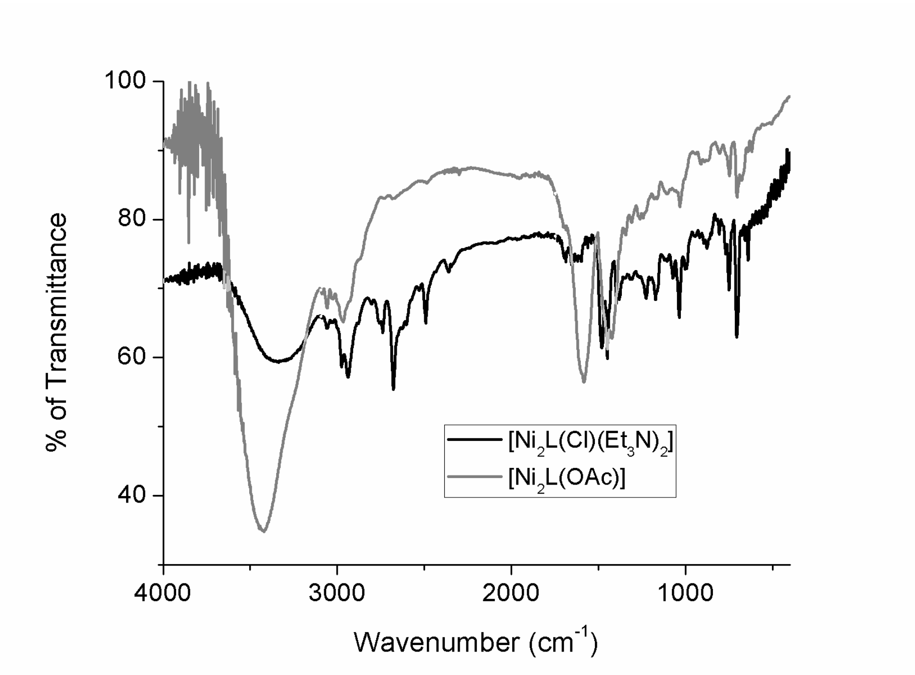

SI1. Solid infrared spectra of [Ni$_2$L(OAc)](gray line) and [Ni$_2$L(Cl)(Et$_3$N)$_2$] (black line) complexes.

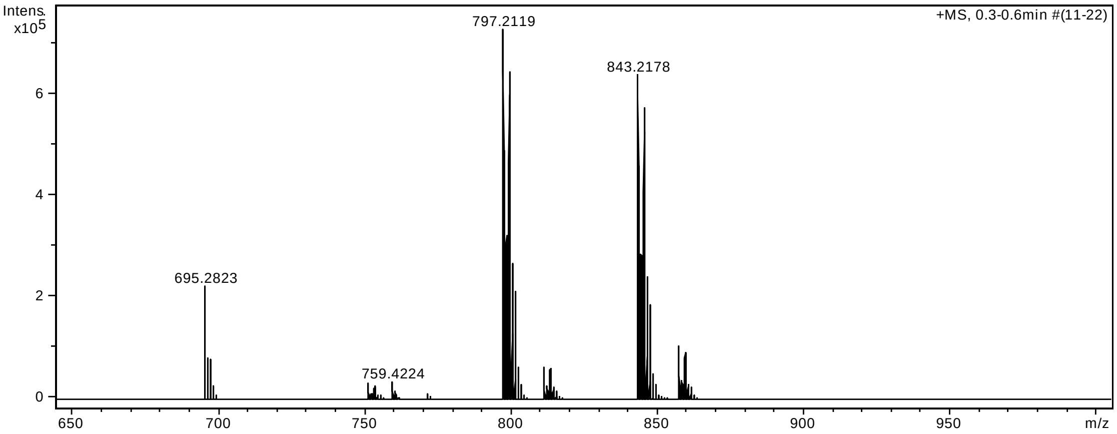

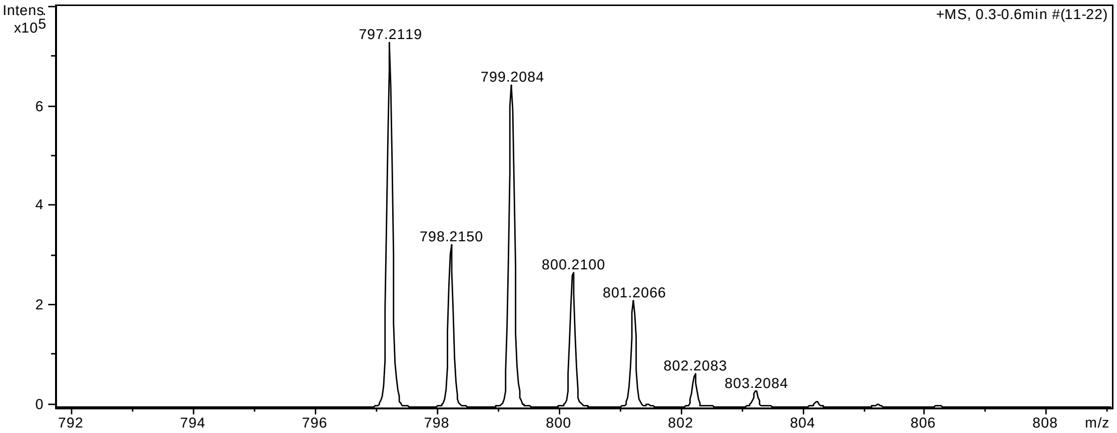

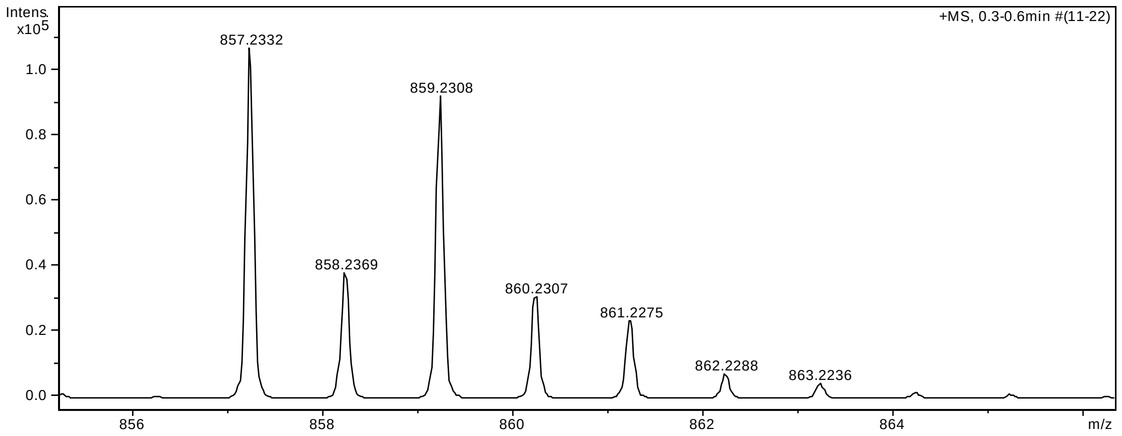

SI2. HRMS of [$Ni_2L(OAc)$].

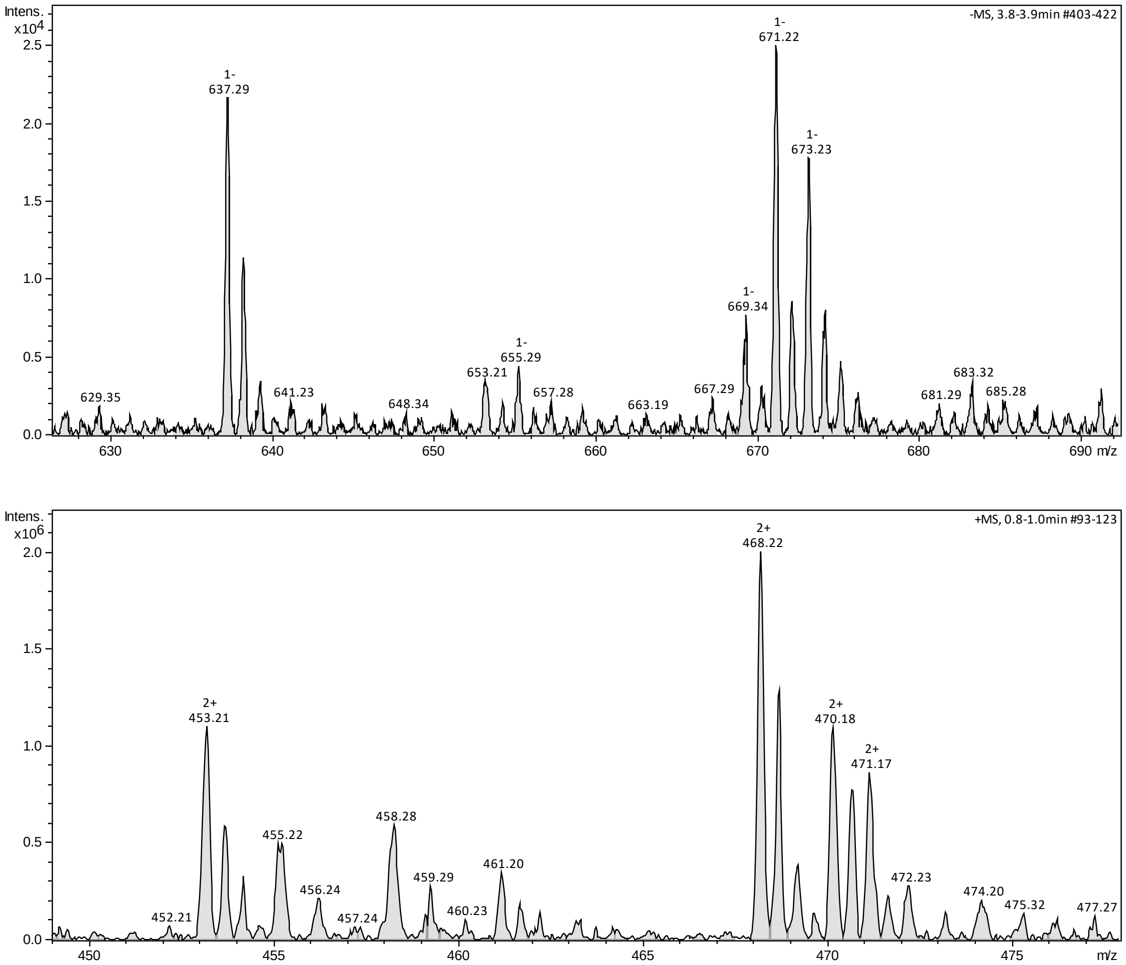

SI3. LRMS of [$Ni_2L(Cl)(Et_3N)_2$]. Negative mode (top panel) and positive mode (bottom panel).

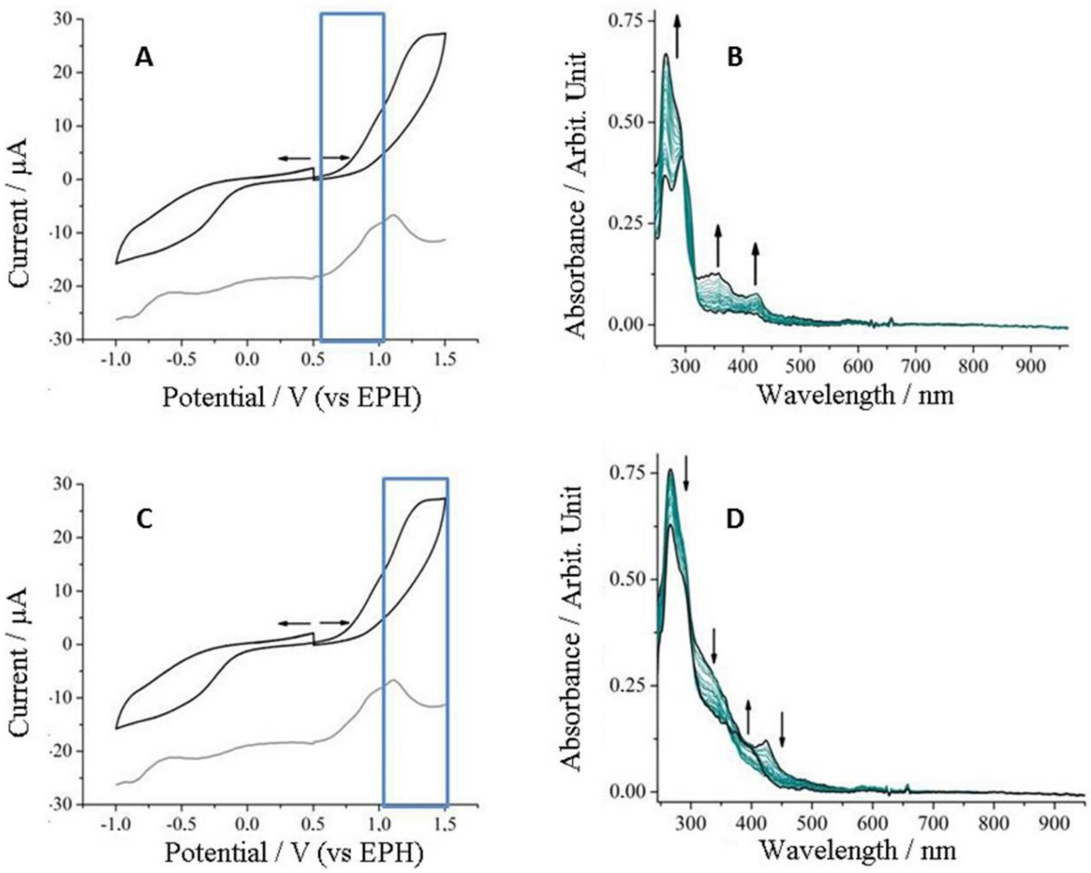

SI4. Anodic cyclic voltammetry and corresponding DPV plot for the complex [$Ni_2L(OAc)$] in DMF and 0.1 M^{-1} $TBAClO_4$ as electrolyte (A and C) and spectrolectrochemistry of the oxidation processes at 0.63V (B) and 1.24V (D).

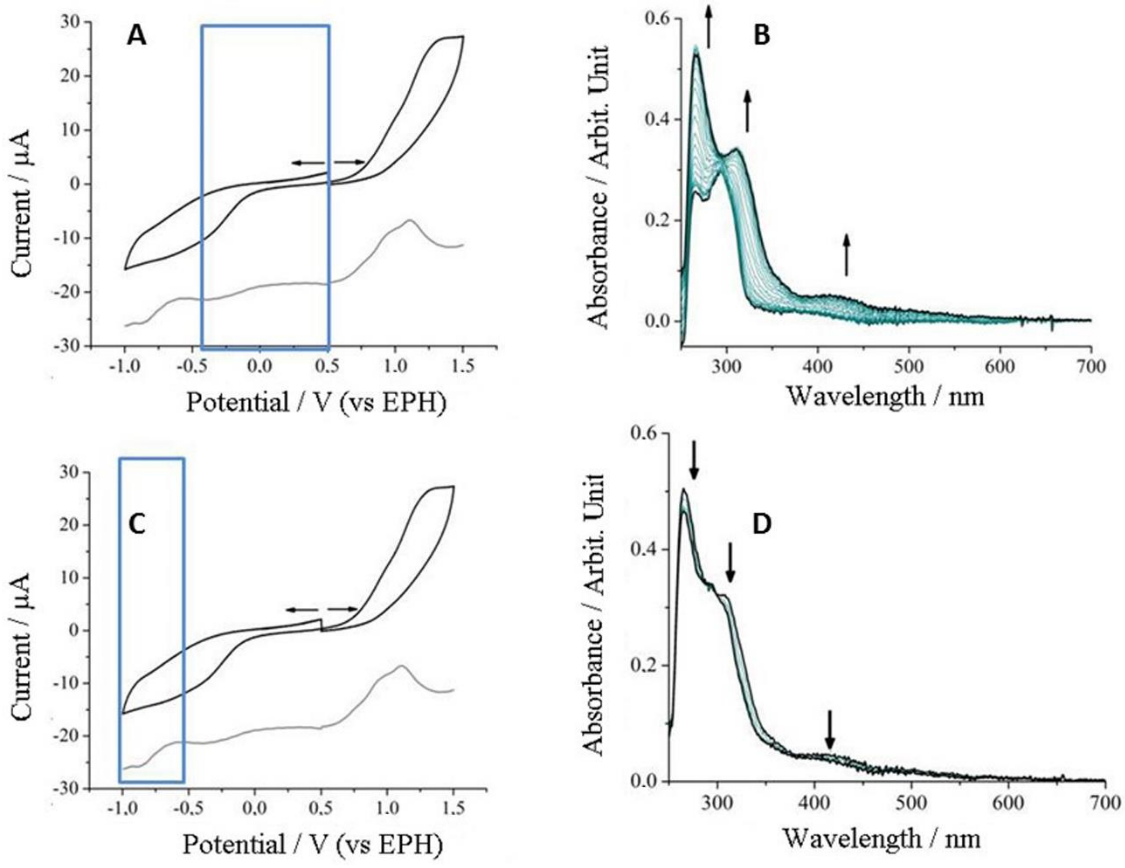

SI5. Cathodic cyclic voltammetry and corresponding DPV plot for the complex [$Ni_2L(OAc)$]in DMF and 0.1 mol L^{-1} $TBAClO_4$ as electrolyte (A and C) and spectrolectrochemistry of the reduction processes at -0,36 (B) and -0,88V (D).

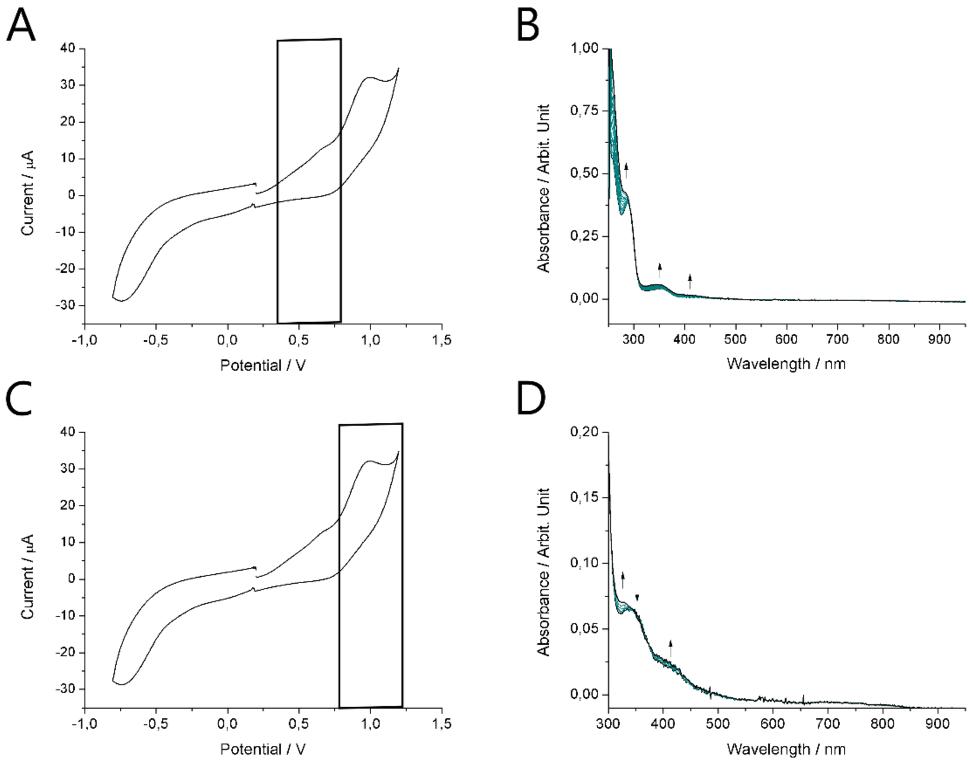

SI6. Anodic cyclic voltammetry and corresponding DPV plot for the complex [$Ni_2L(Cl)(Et_3N)_2$] in DMF and 0.1 M^{-1} $TBAClO_4$ as electrolyte (A and C) and spectrolectrochemistry of the oxidation processes at 0.5V (B) and 0.8 V(D).

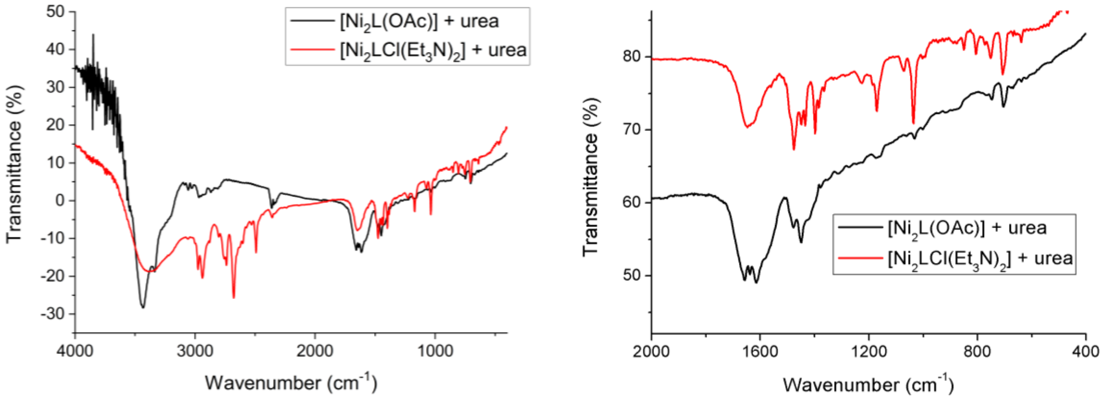

SI7. Infrared of coordinated urea to [$Ni_2L(OAc)$] and [$Ni_2L(Cl)(Et_3N)_2$] complexes. Left: spectra from 4000 to 400cm^{-1} and Right: Spectra from 2000 and 400 cm^{-1}.

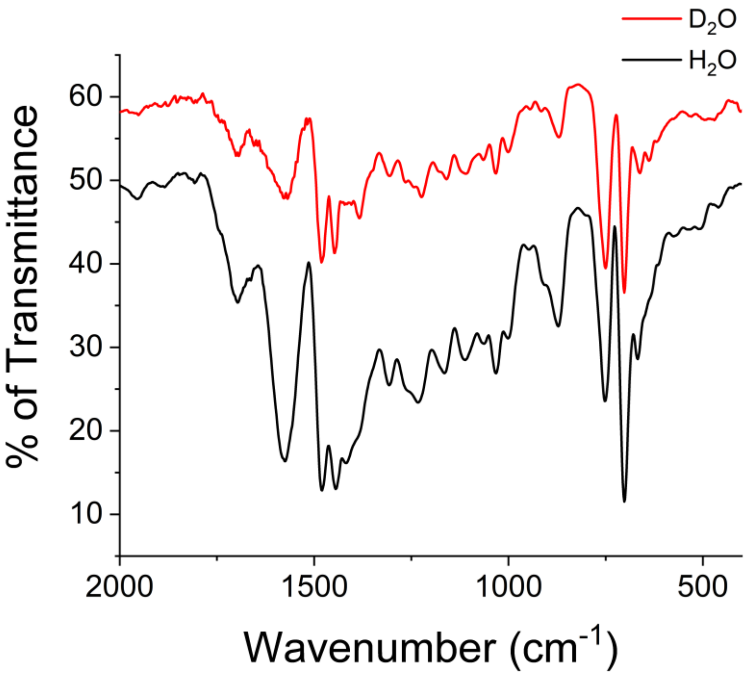

SI8. Infrared of the soluble fraction of the reaction between [$Ni_2L(OAc)$] and water. Black line represents the product obtained after titration with H_2O and red line represents the product obtained after titration with D_2O.

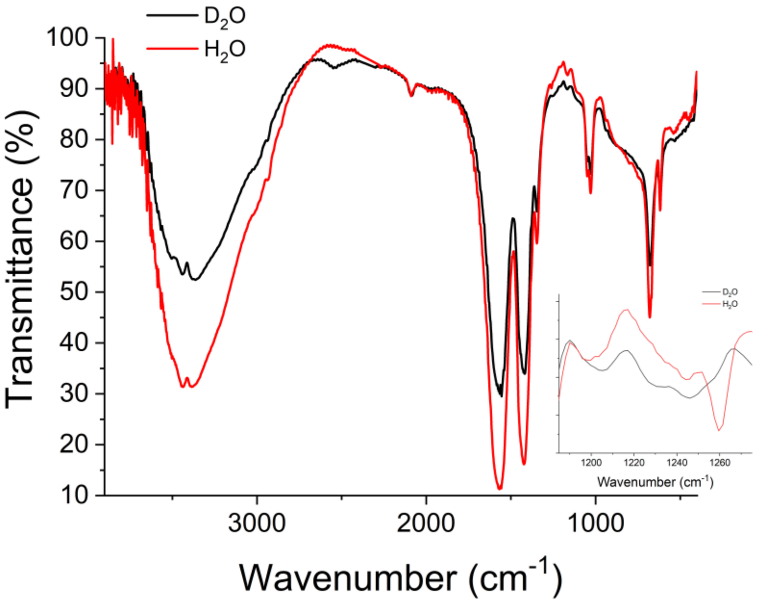

SI9. Infrared of the insoluble fraction of the reaction between [$Ni_2L(OAc)$] and water. Black line represents the product obtained after titration with D_2O and red line represents the product obtained after titration with H_2O. Inset: FTIR spectra between 1185 cm^{-1} and 1275 cm^{-1} .

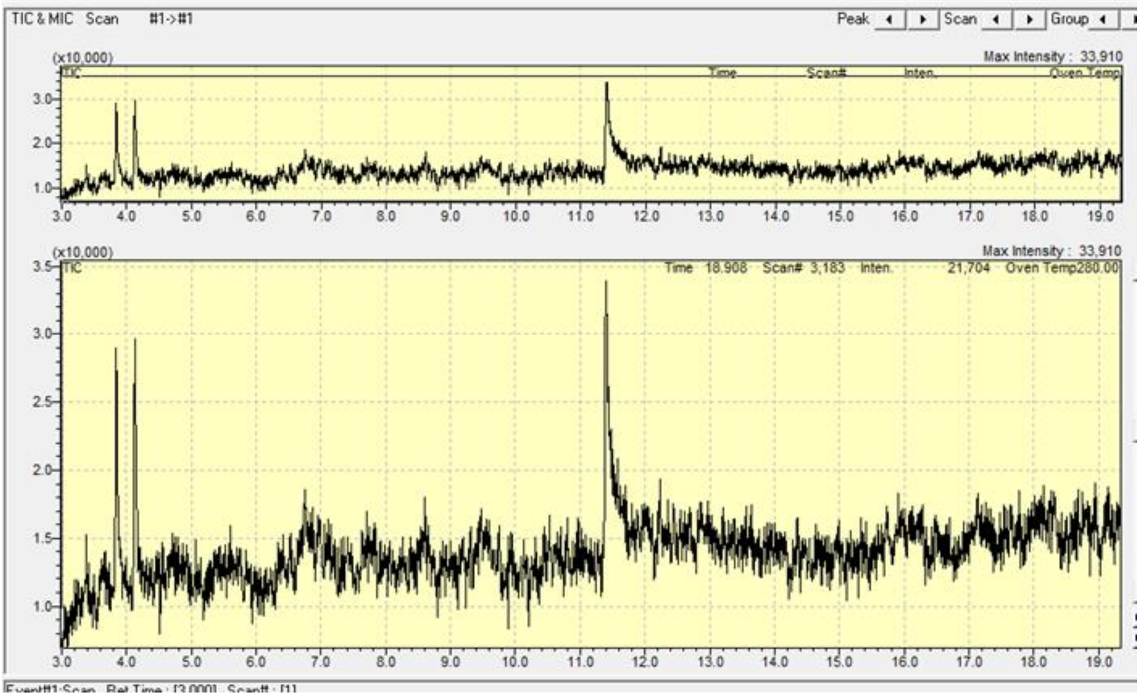

SI10. GC-MS chromatogram of N-phenylurea decomposition using [$Ni_2L(OAc)$] as a catalyst. Phenylisocyanate has a retention time of 3.8 min, aniline has a retention time of 4.1 min and N-phenylurea has a retention time of 11.5 min.

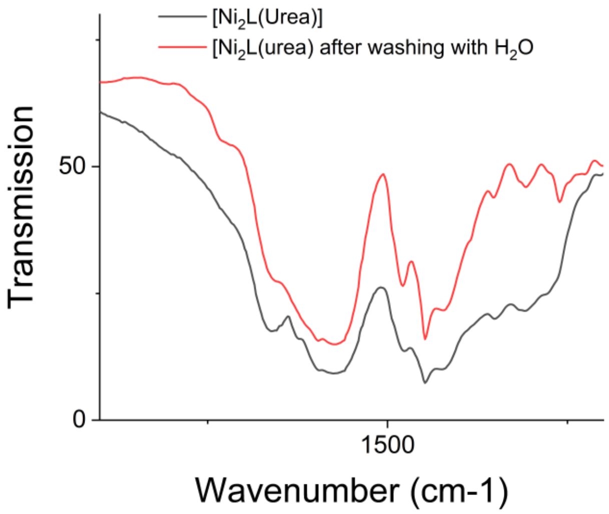

SI11. Infrared of [Ni_2L(Urea)] before (black line) and after (red line) washing the complex with water.
